# Habitat Selection and Post-Release Movement of Reintroduced Brown Treecreeper Individuals in Restored Temperate Woodland

**DOI:** 10.1371/journal.pone.0050612

**Published:** 2012-12-05

**Authors:** Victoria A. Bennett, Veronica A. J. Doerr, Erik D. Doerr, Adrian D. Manning, David B. Lindenmayer, Hwan-Jin Yoon

**Affiliations:** 1 Fenner School of Environment and Society, The Australian National University, Canberra, Australian Capital Territory, Australia; 2 Ecosystem Sciences, Commonwealth Scientific and Industrial Research Organisation, Canberra, Australian Capital Territory, Australia; 3 Division of Evolution, Ecology, and Genetics, Research School of Biology, Australian National University, Canberra, Australian Capital Territory, Australia; 4 Statistical Consulting Unit, School of Mathematical Sciences, The Australian National University, Canberra, Australian Capital Territory, Australia; Australian Wildlife Conservancy, Australia

## Abstract

It is essential to choose suitable habitat when reintroducing a species into its former range. Habitat quality may influence an individual’s dispersal decisions and also ultimately where they choose to settle. We examined whether variation in habitat quality (quantified by the level of ground vegetation cover and the installation of nest boxes) influenced the movement, habitat choice and survival of a reintroduced bird species. We experimentally reintroduced seven social groups (43 individuals) of the brown treecreeper (*Climacteris picumnus*) into two nature reserves in south-eastern Australia. We radio-tracked 18 brown treecreepers from release in November 2009 until February 2010. We observed extensive movements by individuals irrespective of the release environment or an individual’s gender. This indicated that individuals were capable of dispersing and actively selecting optimum habitat. This may alleviate pressure on wildlife planners to accurately select the most optimum release sites, so long as the species’ requirements are met. There was significant variation in movement between social groups, suggesting that social factors may be a more important influence on movement than habitat characteristics. We found a significant effect of ground vegetation cover on the likelihood of settlement by social groups, with high rates of settlement and survival in dry forests, rather than woodland (where the species typically resides), which has implications for the success of woodland restoration. However, overall the effects of variation in habitat quality were not as strong as we had expected, and resulted in some unpredicted effects such as low survival and settlement in woodland areas with medium levels of ground vegetation cover. The extensive movement by individuals and unforeseen effects of habitat characteristics make it difficult to predict the outcome of reintroductions, the movement behaviour and habitat selection of reintroduced individuals, particularly when based on current knowledge of a species’ ecology.

## Introduction

Species reintroduction programmes aim to re-establish a population of a locally-extinct species within its historical range [1]. Reintroductions are an increasingly important and effective tool to counter biodiversity loss and conserve threatened species [2], [3], [4], [5]. However, reintroductions are not always successful [3], [4], [6]. The success of a program is often dependent upon the suitability of the habitat at the release site [4], [6], [7], [8]. Therefore, there is considerable benefit in not only ensuring that the habitat quality at the release site is adequate, but also monitoring the survival, movement and habitat selection of released individuals, particularly using an experimental approach to examine the effect of applied habitat treatments [9], [10].

The habitat quality at a release site is likely to influence the movement of released individuals. In particular, individuals released in poor quality habitat may be more inclined to disperse to search for better quality habitat [11], [12]. Variations in habitat quality have been shown to influence the dispersal strategies of both reintroduced species [9] and natal dispersers [13], [14]. Other factors potentially influencing the choice to leave an area include local population density, age, reproductive status, body condition and predation pressure [15], [16], [17]. Dispersal away from a release site following reintroduction also may be influenced by the translocation process, releasing individuals within an unfamiliar environment and experiences in the natal habitat [16], [18]. As a result, some released individuals may move rapidly away from a release site [19], [20], move greater distances than is usually recorded for the species [21], [22], [23], or even attempt to return to the home capture site [18], [24].

Classic optimal habitat choice models suggest that dispersing individuals, and indeed reintroduced individuals, will settle within optimal habitat rather than sub-optimal habitat [25]. Information drawn from the movement and eventual habitat selection of reintroduced individuals can provide insights into how animals perceive their environment. In particular, monitoring released individuals can confirm, or falsify, hypotheses about patterns of habitat selection for a species. Additionally, we can gain insights into the species’ ability to search the environment to locate high quality habitat, the costs of moving through an unfamiliar environment (such as difficulty in locating food, increased predation rates and a lack of knowledge of escape routes from predators [26], [27], [28], [29]), and the potential influences of choices on survival, and hence reintroduction success.

To test hypotheses about habitat selection, we reintroduced seven social groups (43 individuals) of the brown treecreeper (*Climacteris picumnus*), into Mulligans Flat and Goorooyarroo Nature Reserves in the Australian Capital Territory from 16 November to 1 December 2009 [30]. These temperate woodland reserves are managed as a large-scale experimental restoration project [31], [32]. Restoration treatments and controls were applied across the reserves, including maintaining differences in the level of vegetation cover in the ground layer. Prior to the reintroduction, species-specific nest-boxes were installed as an additional experimental treatment. Previous research has suggested that the vegetation structure of the ground layer and density of tree hollows are two of the most important factors influencing the presence and reproductive success of the species [33], [34]. The integration of an experimental framework into the programme allowed for the unique examination of how habitat variation influenced the movement, habitat choice and survival of reintroduced individuals. In particular, we monitored reintroduced brown treecreeper individuals to test five key hypotheses:

1. Individuals actively search for good quality habitat so that even in the absence of competition they will still explore the wider environment before choosing where to settle. Thus, movement paths will show a decrease in search area over time until a minimum threshold, or asymptote, is reached.
2. Individuals may search less widely when released in higher quality rather than in lower quality habitats, with habitat quality predicted a priori from previous ecological studies (i.e. higher quality habitat was woodland areas with lower ground vegetation cover, which is based on the species’ preference for foraging on the ground [35], [36], [37] and that low ground vegetation may allow for increased accessibility to invertebrate prey and easier escape from predators [34], [38], and also areas with nest-boxes installed, which may provide an escape hollow when under threat or a roosting site [39], [40]).
3. Based on the classic optimal habitat choice models [25], individuals in restored environments will settle in the highest quality habitat that they encounter during the search phase.
4. The habitat types (in terms of the experimental treatments) that are used most by released brown treecreepers will influence survival.
5. Groups that take a greater time to search and settle in an unfamiliar environment will have reduced short-term survival in comparison to social groups that settle earlier.

Tests of these five hypotheses will provide a greater understanding of how reintroduced individuals move through their release environment, the importance of habitat quality at the release site, and provide implications for how we predict habitat quality. This is particularly important given the growing prevalence of reintroductions to combat biodiversity loss [2], [41].

## Methods

### Ethics Statement

This study was conducted in strict accordance with animal ethics approval obtained through The Australian National University Animal Experimentation Ethics Committee (C.RE.55.08). All reasonable actions were taken to minimise the impact on the welfare of the animals involved, including utilising appropriate methods for the capture, transport and monitoring of reintroduced brown treecreepers.

The project was conducted under a New South Wales Office of Environment and Heritage Scientific Licence (S12906) and Export Licence (IE095650); and a Licence to Import from the Australian Capital Territory Department of Territory and Municipal Services (LI2008330). Accessed land was a mixture of private property, travelling stock reserves managed by the Hume Livestock Health and Pest Authority and Nature Reserves managed by the Australian Capital Territory Department of Territory and Municipal Services.

### Study Area

**Figure 1:**
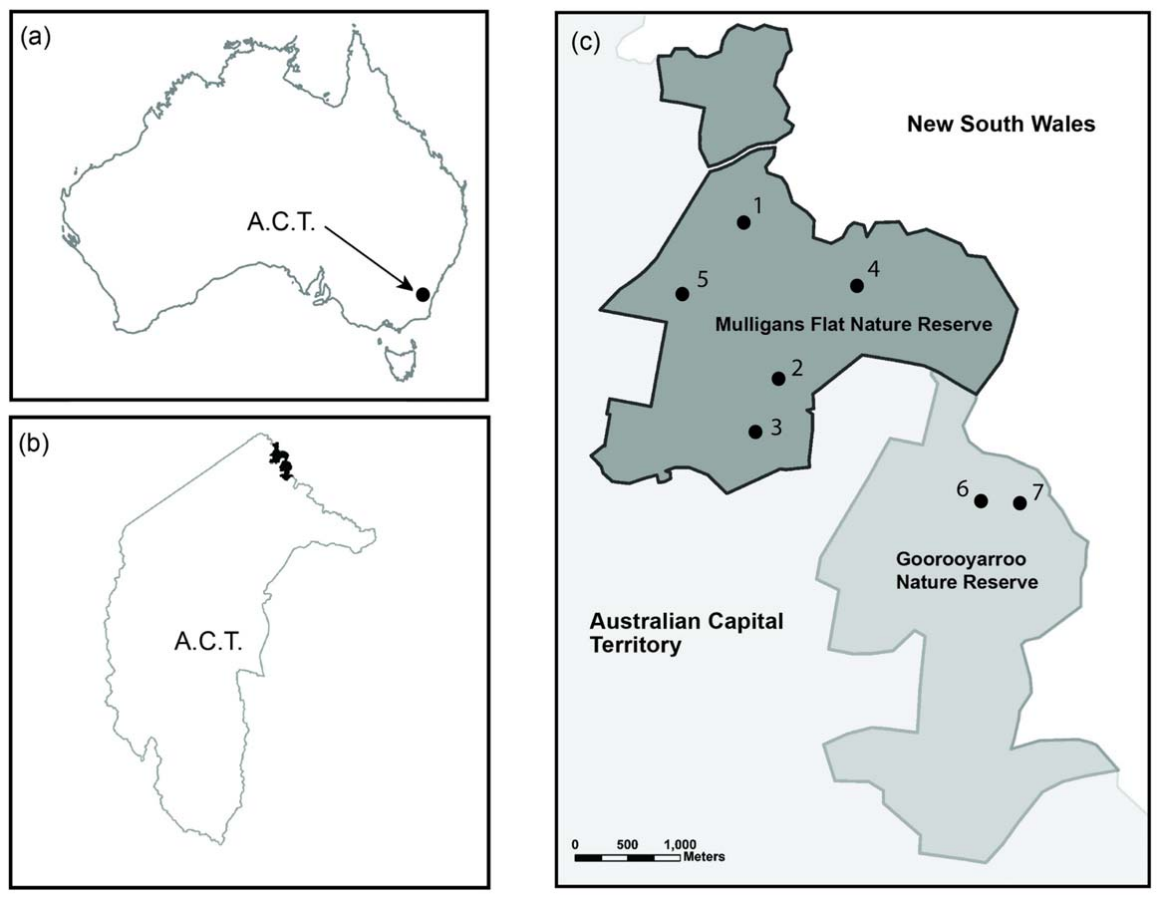
Mulligans Flat and Goorooyarroo Nature Reserves. The location of Mulligans Flat and Goorooyarroo Nature Reserves in northern Australian Capital Territory (ACT) including: (a) The location of the ACT within Australia; (b) The nature reserves within the ACT; and (c) The release locations for the seven Brown Treecreeper social groups.

We conducted this study at Mulligans Flat Nature Reserve and Goorooyarroo Nature Reserve, in north-eastern Australian Capital Territory, in south-eastern Australia (Figure 1). In total, the reserves cover 1623 ha of partially-modified, lowland temperate woodland and dry forest [31], [32]. The reserves are the location of the ‘Mulligans Flat – Goorooyarroo Woodland Experiment’ [31], [32] and were previously stratified into ‘polygons’ according to vegetation type and structure. We then selected twenty-four polygons containing woodland as experimental polygons (ranging from 9.92 to 90.08 hectares, average 26.09 (±3.43 s.e.) hectares). We utilised these experimental polygons for analysis of how brown treecreeper movement, survival and habitat selection varied in relation to the experimental treatments. We classified each of the experimental polygons according to two experimental treatments: (1) high or medium ground vegetation cover; and (2) the presence or absence of artificial nest boxes.

We assigned a category for ground vegetation cover to each experimental polygon using data on vegetation characteristics collected by McIntyre *et al.* [42]. We extracted their data on total biomass and live plant basal area of all herbaceous plants plus sub-shrubs <50 cm tall for each polygon. Following this, we created standardised scores (Student’s t-statistic, i.e. z-scores for a population that has only been sampled and is not fully known) for each of these variables and summed the scores to create a standardised measure for each polygon. The measure incorporated both basal area and biomass because both could influence the quality of the ground layer and the ability of brown treecreepers to manoeuvre while ground-foraging. We then ranked the experimental polygons, with the lower 50% classified as containing ‘medium’ amounts of ground vegetation cover and the upper 50% were classified as containing ‘high’ ground vegetation cover. As brown treecreepers also utilized areas that were outside the experimental polygons (both during dispersal and after settlement), we classified non-experimental woodland areas as medium or high ground vegetation cover through comparison with experimental polygons. If an area was dry open forest, we assigned it a ‘low’ level of ground vegetation cover, since Australian dry open forest typically contains a greater density of trees than woodland, which is associated with a lower level of ground vegetation cover [43], [44].

We installed two hundred and sixteen species-specific nest boxes within half (12) of the experimental polygons, half in polygons with medium ground vegetation cover and half in polygons with high ground vegetation cover. We clustered the nest boxes within large trees (four or five per tree) to make them more apparent to the brown treecreeper. Polygons that received nest boxes were distributed relatively uniformly across the two nature reserves such that they were not all clustered in a small area. We designed the nest boxes using knowledge of the behaviour and natural nesting hollow dimensions of the brown treecreeper [40].

### Study Species

The brown treecreeper is a facultative cooperative breeder, living predominantly in gregarious social groups comprised of a breeding pair and a number of offspring that have delayed dispersal [45]. Females disperse earlier and further than males, with dispersal averaging 1.14±1.25 km with a maximum of 4.5 km [46], while males generally disperse no further than an adjacent territory (<500 m) [45]. Social groups occupy territories averaging 3–6 ha in size, ranging to as much as 10.7 ha in lower quality habitat [33], [45]. The brown treecreeper nests and roosts in naturally-occurring tree cavities in a variety of eucalypt species [47] and is a ground and bark-foraging insectivore [36], [37].

Currently, there is evidence of dramatic declines in brown treecreeper population density as well as extinction of local populations over many areas [35], [48]. The main causes of decline for the brown treecreeper include fragmentation (due to the species’ short-distance dispersal characteristics) [35], [45], [46], and habitat degradation such as the loss of tree hollows [33], coarse woody debris and ground litter [37], [49], and alterations in ground vegetation density [34]. Thus, the restoration treatments described above were specifically thought to address the likely causes of local decline for the brown treecreeper and recreate habitat suitable for this species [30]. Reintroduction was deemed necessary as the species’ limited dispersal distances, and the lack of an existing population of the species within 15 km, are thought to make natural recolonisation of these reserves extremely unlikely. Further, the brown treecreeper is a member of a suite of woodland birds thought to be most sensitive to decline [48], [50], and hence the results of this study are likely to be applicable to other ground-foraging insectivores.

### Translocation and Radio-telemetry

We captured social groups from wild source populations located approximately 200 km west of the release sites, south-east of Wagga Wagga, New South Wales. The brown treecreepers that we translocated were captured from populations that had been studied since September 2005, with the majority of individuals colour-banded and the social relationships already documented [51]. We attempted to capture entire social groups for translocation, however in some cases we failed to capture some helpers (although the breeding pair were always captured), who then remained in the source populations. Members of a social group can be determined since individuals predominantly interact with members of their own group and females generally remain territorial [52]. Although, males may feed at nests within neighbouring territories that contain related males during the breeding season [53]. We released brown treecreeper social groups (adult breeders, adult non-breeders or helpers, and dependent fledglings) sequentially, approximately every second day from 16 November to 1 December 2009 (Information S1). Each group (four to eight individuals) was released in a unique polygon representing a combination of the experimental treatments (level of ground vegetation cover and presence or absence of nest boxes). We attempted to replicate each treatment combination twice, resulting in a total of eight groups. However, for logistical reasons only seven groups were captured.

We fitted 18 adult brown treecreepers (at least two individuals per social group) with radio-transmitters (Holohil Systems Model BD-2, weight 0.9 g or 2.8% of the average bird weight). Radio-transmitters of this kind have been used extensively in brown treecreeper studies in the past [51], [54]. We radio-tracked individuals daily from release in November 2009, until 4 February 2010 with generally at least twice-daily fixes to record the global position of individuals (UTM coordinates). However, we also performed more frequent checks of birds’ radio-transmitter signals throughout the day which allowed us to determine whether a bird had moved to a different area or not a minimum of four times each day (twice in the morning and twice in the afternoon). When such checks suggested that an individual bird had moved from the general vicinity of its previous location, we physically located the bird to record additional fixes. We obtained as many locations as possible for all radio-tracked individuals until the battery of the radio-transmitter failed or the individual died or disappeared. We were then able to connect consecutive fixes (locations) for each individual to approximate their movement path. This protocol was designed to capture all exploratory movements based on knowledge of the approximate duration and timing of exploratory forays made by dispersing brown treecreepers. We developed the protocol through prior extensive radio-tracking of dispersing brown treecreepers in both continuous and fragmented landscapes [54], [55].

It has been recommended that animal locations be separated by sufficient time (e.g. the time-to-independence) to eliminate autocorrelation bias [56], [57]. The time-to-independence is an estimate of the time required for an animal to traverse its home range [57], [58], which for the brown treecreeper is often only 15 minutes. Our sampling of brown treecreeper locations were always at least 15 minutes apart (and often hours apart), which should eliminate autocorrelation. Further, recent studies have indicated that the focus on time-to-independence is flawed, and biologically important information can be gained from observations taken close together [56], [57], such as a closer approximation of an individuals’ movement path.

### Examination of Search Techniques

To determine whether released brown treecreeper individuals actively searched for good quality habitat, we examined their exploratory forays and analysed the individuals’ movement paths. Brown treecreeper natal dispersers use a foray-based search strategy, usually originating from the home territory, to locate breeding territory vacancies, where they eventually settle [54]. As we expected, reintroduced brown treecreeper individuals used similar behaviours to find habitat to settle in. They focused their activities within temporary home ranges, usually initially around or close to the release site, then used exploratory forays to find and move between temporary home ranges until eventually settling in a final territory. We thus developed a foray identification technique to distinguish exploratory forays from the initial, temporary home range (Information S2). This technique distinguished larger foray movements from those within the range of a normal home territory. Using this technique, we then calculated four movement parameters to describe different aspects of the movement paths of each individual: (1) foray distance; (2) foray rate; (3) search rate; and (4) search area. These parameters were based upon existing studies of brown treecreeper dispersal from natal territories [51], [54]. We calculated foray distance by adding the lengths of all forays for each individual bird. We determined foray rate as the number of forays divided by the number of days tracked per bird. We calculated search rate by dividing the total length of the movement path for each individual by the total number of location points recorded for that bird. Finally, we calculated search area using the assessment corridor method in the program DRAP v0.99 as described in Doerr and Doerr [54]. This method widens the movement path based on the distance over which an individual is likely to be able to assess all aspects of habitat quality as they move through the habitat, termed the ‘assessment radius’, which we set at 50 m. The 50 m distance was chosen based on prior observations of response to habitat features and an approaching observer [54]. This approach has been used successfully in other studies and one key point is that the results are used in a relative sense, to compare among individuals, so the precision of this estimate is not critical [59]. Subsequently, we defined the assessment corridor (or search area) as the total area covered by this widened search path. We conducted analyses on search rate and search area for all radio-tracked birds. However, analyses on foray distance and foray rate were conducted only on birds for which we could distinguish forays based on the foray identification technique (Information S2).

We further examined brown treecreeper search movement to determine whether movement paths show a threshold-like decrease over time as individuals established a home range territory. To do this, we calculated the search area of the movement path for each individual on a weekly basis using the assessment corridor method detailed above.

### Habitat Attributes at Settlement

To determine whether brown treecreeper social groups settled in the highest quality habitat they encountered, we determined the location point at which a social group had settled (see Information S3 for technique). By identifying the date that this location point was recorded, we could then also determine the time taken to settle for each group. We established the home range for a social group by creating a minimum convex polygon around the locations recorded after a group had settled using ESRI® Arcmap™ 9.2. We then determined the habitat characteristics of a social group’s final home range according to the level of ground vegetation cover and the presence or absence of nest boxes in the constituent polygon(s). When the final home range overlapped more than one polygon, the social group was determined to have settled in a polygon (i.e. used it as part of their home range) if ≥25% of locations after settlement were within that polygon.

### Habitat Effects on Survival

To examine the influence of the habitat type most used on survival, we monitored the survival of reintroduced brown treecreeper individuals on a daily basis throughout the radio-tracking period (to 4 February 2010). This was followed by monthly monitoring for survival from March 2010 to March 2011. This involved targeted searches to locate and identify individuals known or suspected to be alive each month. Survival was assessed as the number of days (or months for monthly survival) that an individual was confirmed alive after release, with disappearances treated as non-survival. This method accounted for the staggered release of social groups. We identified the habitat characteristics most experienced by a radio-tracked individual (in terms of the level of ground vegetation cover (low, medium or high) and the presence or absence of nest boxes) over the daily radio-tracking period and during monthly surveying. This was determined by calculating the number of times that the individual was located in areas with each of the habitat characteristics. Hence, the habitat characteristics with the highest number of locations for that individual were the characteristics most experienced.

### Costs of Searching in Unfamiliar Habitat

To examine the effect of the time taken to search and settle in an unfamiliar environment on short-term survival, we determined the number of individuals alive at settlement for each group that settled, out of the total number of individuals released.

### Statistical Analysis

We only obtained data for statistical analysis from radio-tracked brown treecreepers. To quantify how widely individuals searched the reserves for habitat in which to settle, we calculated the four movement parameters (foray distance, foray rate, search rate and search area) and summarised them using descriptive statistics. We then analysed whether individuals decreased their extent of search over time using a linear mixed model (LMM) [60] to examine the effect of monitoring week on the weekly search area (unit of analysis = bird-week, n = 106). We included only data obtained from monitoring individuals over complete weeks (i.e. seven days for each week) and only for individuals with at least two weeks of radio-tracking data. We used log transformations to achieve normality of search area and incorporated gender as a covariate. We included “individual bird” as a random factor in the model due to the repeated data collection from each individual that was assessed. The relationship between monitoring week and weekly search area appeared to approximate a quadratic relationship for some birds. Therefore, we fitted a quadratic regression model.

The unit of analysis for all other analyses about movement was the individual bird (n = 18) and all predictor variables examined applied to individuals. However, data were nested such that individual birds (level-1 units) were clustered within social groups (level-2 units). This data structure does not indicate pseudoreplication, as the predictor variables vary with level-1 units rather than level-2 units. However, the influence of the level-2 units needs to be taken into account. Modern approaches that avoid data averaging and allow researchers to take full advantage of the sample size of level-1 units include hierarchical modelling or mixed effects modelling using restricted maximum likelihood procedures [61]. We used the latter, modelling level-2 units (social groups) as random effects in analyses that were conducted at the individual (n = 18) level.

To examine whether the habitat quality at the release site affected how extensively individuals searched the reserves, we analysed the relationships between the characteristics at an individual’s release site (level of ground vegetation cover and presence or absence of nest boxes) and the four movement parameters (foray distance, foray rate, search rate and search area). We constructed LMMs for each of the movement parameters, following log transformation of the data on search rate and foray distance to achieve normality. We included gender of the individual as a covariate. Since not all radio-tracked individuals were sampled with equal effort (due to increased sampling effort to record unusual dispersal movements and differential length of transmitter battery life), we adjusted our calculations of search area to attain consistency between individuals. Search area would be expected to increase with an increase in the number of locations for individuals actively searching their environments for habitat. We confirmed this by plotting search area against number of locations, which revealed a roughly linear relationship. Thus, we divided search area for each individual by the number of locations for that individual to obtain an estimate of area searched per location obtained. We included social group as a random factor in the LMMs as the movement of one individual may be influenced by the movement of other group members.

To determine whether individuals settled in the highest quality habitats that they encountered based on our a priori understanding of habitat quality, we constructed a contingency analysis for each of the experimental treatments (ground vegetation cover and nest boxes) separately using SYSTAT 10 (SPSS Incorporated, Chicago, Illinois, USA). The two contingency analyses tested for association between whether brown treecreeper social groups settled within a polygon or not with either: (1) the level of ground vegetation cover within a polygon (low, medium, or high); or (2) the presence or absence of nest boxes. We included only the polygons where brown treecreeper social groups were observed through radio-tracking in the analysis (i.e. habitat selection was analysed relative to habitat experienced rather than habitat available). For the analysis involving nest boxes, we used the Fisher’s exact test and excluded all polygons with low levels of ground vegetation cover as none of these polygons received nest boxes. For the analysis of the effect of ground vegetation cover, we used the Chi-square likelihood ratio.

We examined the influence of the habitat experienced while searching on survival by separating the data on survival of radio-tracked individuals into: (1) the number of days known to be alive during the radio-tracking period only (16 November 2009 to 4 February 2010); and (2) the number of months known to be alive from release until March 2011. For consistency in detectability, we only analysed survival for individuals released with radio-transmitters attached. We log-transformed daily survival to achieve normality and the data were analysed using a generalised LMM. We analysed monthly data with a Poisson distribution since the response variable was in the form of counts data. Our analyses examined the relationship between survival and the habitat characteristics most experienced by each individual in terms of the level of ground vegetation cover. Brown treecreeper individuals were seen predominantly in polygons containing no nest boxes; therefore there was insufficient variation to include this factor in the analyses. We included bird gender and social group as fixed and random factors respectively.

To analyse the relationship between time taken to settle and survival, we employed a generalised linear regression using binomial distribution. Our analyses examined the influence of a social group’s time to settlement on the number of individuals in that social group alive at settlement. The total number of group members at release was set as the binomial total.

We examined the significance of random factors for all relevant analyses using a likelihood ratio test, which compared the deviances (2 times the log likelihood) of models with and without the random factor included [62], [63]. If removing the random factor caused a large enough drop in the log-likelihood, when compared to a chi-squared distribution with degrees of freedom equal to the number of additional models in the more complex model, then the factor was statistically significant. If the difference was not significant, we eliminated the random factor and general linear models were constructed [64]. For models containing two or more independent fixed variables, we used backward elimination to remove the least significant variables from the model using the Wald statistic. We continued this until all variables in the final model were statistically significant (P<0.05). We used this method since it is a standard statistical test for comparing nested models [62], [65], the experimental treatments were guided by the clear development of hypotheses, and the number of variables was small enough to consider all possible models (full model vs. possible nested models).We conducted all statistical analyses using Genstat (13th edition, VSN International, Hemel Hempstead, UK) except where specified.

## Results

We recorded a total of 1447 locations for 18 radio-tracked brown treecreeper individuals from 16 November 2009 to 4 February 2010. The average number of locations per bird was 80.39 (±12.28 s.e.), and ranged from four to 157 locations. We tracked individuals for an average of 43.33 (±6.01 s.e.) days resulting in an average total distance moved of 13.65 (±3.27 s.e.) kilometres. Large variations in the values listed above were due to some individuals losing their radio-transmitter early (i.e. before 9 weeks of use), or early fatality.

After release, six of the social groups left their release polygon and began exploring the wider environment. There was not enough data collected on the seventh group to determine their movements due to early fatalities and the loss of radio-transmitters. We observed some groups moving relatively linearly (i.e. not in forays) as a unit and settling in new areas. In comparison, we also observed a number of instances of individuals moving independently rather than as a group. This included some breeding females moving away from fledglings for some time to conduct forays (the majority of these forays took less than one day, but up to three days). These dispersal movements often resulted in the entire social group eventually moving to new locations. There was only one occasion where an individual undertook a foray and settled independently away from its social group.

### Search Techniques

Of the 18 radio-tracked brown treecreeper individuals, we observed seven (39%) embarking on forays (Table 1). The two individuals with the highest foray distances and two of the three highest foray rates (KGMG and UBMR) were members of the two social groups released within Goorooyarroo Nature Reserve. The search rate for all radio-tracked brown treecreeper individuals averaged 143.76 metres/location (±20.75). The average search area for all individuals was 77.47 ha (±19.36 s.e.), ranging up to 288.84 ha.

**Figure 2:**
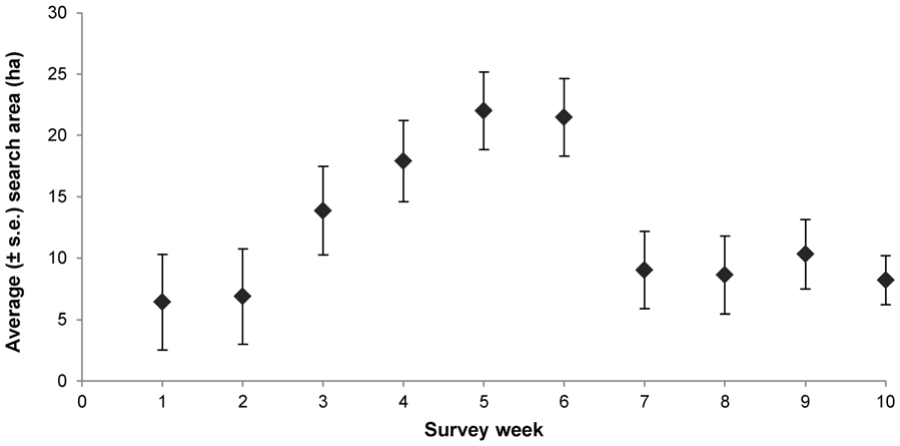
Weekly search area. The average (± s.e.) search area in hectares for radio-tracked brown treecreeper individuals on a weekly basis. The number of individuals included in the analysis of search area per week are (from week 1 to 10): 15, 15, 13, 11, 10, 10, 10, 10, 8 and 4; total n = 106.

**Table 1:** Search patterns and movement parameters.

Bird ID	Sex	Socialgroup	Number offorays	Range of foraydistances (m)	Total distanceof forays (m)	Foray rate(foray/day)	Furthest distance (m)
GLMU	F	2	1	3627	3627	0.014	2051
KGMG	F	6	24	384–5063	30497	0.364	2425
RGMB	M	6	4	908–2880	6000	0.133	907
RUMK	M	4	7	411–6834	17439	0.108	4846
UBMR	M	7	16	445–7599	31227	0.242	3735
USMB	M	4	14	848–2733	18566	0.250	1559
YKMU	F	4	10	347–3736	10498	0.172	1264
**Average (± s.e.)**			**10.86 (±2.96)**	**1550.71 (±168.07)**	**16836.29 (±4166.61)**	**0.18 (±0.04)**	**2398.10 (±537.05)**

Details of the search patterns and movement parameters displayed by the seven adult brown treecreeper individuals that embarked on forays. Details include the number of forays, range of distances of forays, total distance of all forays for that individual, foray rate (number of forays divided by number of days tracked) and the furthest distance from the release site that the individual was recorded.

We identified significant variation in search area among the individuals (σ^2^ = 0.067; P<0.001). However, there was no significant effect of week (χ^2^ = 2.79, d.f. = 1, P = 0.095) or week^2^ (χ^2^ = 2.95, d.f. = 1, P = 0.086) on search area, although examination of the raw data suggested a quadratic relationship between week and search area (Figure 2). There also was no significant effect of gender (χ^2^ = 0.36, d.f. = 1, P = 0.549) on search area.

We examined the influence of the release site habitat characteristics on the extent of released individuals’ movement. The foray distance travelled by individuals was not influenced by the release site characteristics or the gender of the individual (Table 2). Similarly, these factors did not significantly influence brown treecreeper foray rate, search rate or search area. For all of these analyses, significant variation between groups was identified (Table 2).

**Table 2:** Influences on movement parameters.

Response Term	Factor	?^2^	d.f.	P
**Foray distance**	Ground vegetation cover	0.06	1	0.802
	Nest box	1.08	1	0.299
	Gender	1.75	1	0.186
	Social group (σ^2^ = 6.040)			**0.005**
	Error (1.06±0.61)			
**Foray rate**	Ground vegetation cover	0.01	1	0.918
	Nest box	0.90	1	0.343
	Gender	0.82	1	0.364
	Social group (σ^2^ = 0.019)			**0.019**
	Error (0.01±0.00)			
**Search rate**	Ground vegetation cover	0.94	1	0.331
	Nest box-	0.01	1	0.924
	Gender	0.28	1	0.595
	Social group (σ^2^ = 0.092)			**0.027**
	Error (0.05±0.02)			
**Search area**	Ground vegetation cover	0.37	1	0.543
	Nest box	0.05	1	0.826
	Gender	0.07	1	0.785
	Social group (σ^2^ = 0.302)			**0.007**
	Error (0.11±0.05)			

Results from linear mixed models analysing the effect of gender, the level of ground vegetation cover and the presence or absence of nest boxes at the release site on foray distance, foray rate, search rate and search area. Social group had a significant effect in all analyses.

### Habitat Attributes at Settlement

We observed six of the seven groups settle and establish home ranges after release. The time to settlement ranged from five to 45 days, with an average of 28.33 (±5.78 s.e.) days. The home range of the six groups after settlement ranged from 2.64 ha to 29.30 ha, with an average of 12.66 (±4.51 s.e.) hectares. The polygons in which social groups settled averaged 17.89 (±4.60 s.e.) hectares, ranging from 8.66 to 51.58 ha. The home range of all social groups overlapped at least two polygons.

We examined the effect of experimental treatments on whether a polygon was settled in or not (i.e. whether it was used as part of the final home range). We detected a significant effect of the level of ground vegetation cover on settlement (χ^2^ = 6.031, d.f. = 2, P = 0.049). Dry forest polygons with low vegetation cover had the highest proportional rate of settlement (54.55%, n = 11) followed by high and medium polygons (42.86%, n = 3 and 12.50%, n = 2 respectively). We detected that the brown treecreeper utilised 10 polygons with nest boxes, but settled in none of them, and 24 polygons without nest boxes and settled in 11 (46%) of these, a significant difference (Fisher’s exact test: P = 0.046).

### Habitat Effects on Survival

**Figure 3:**
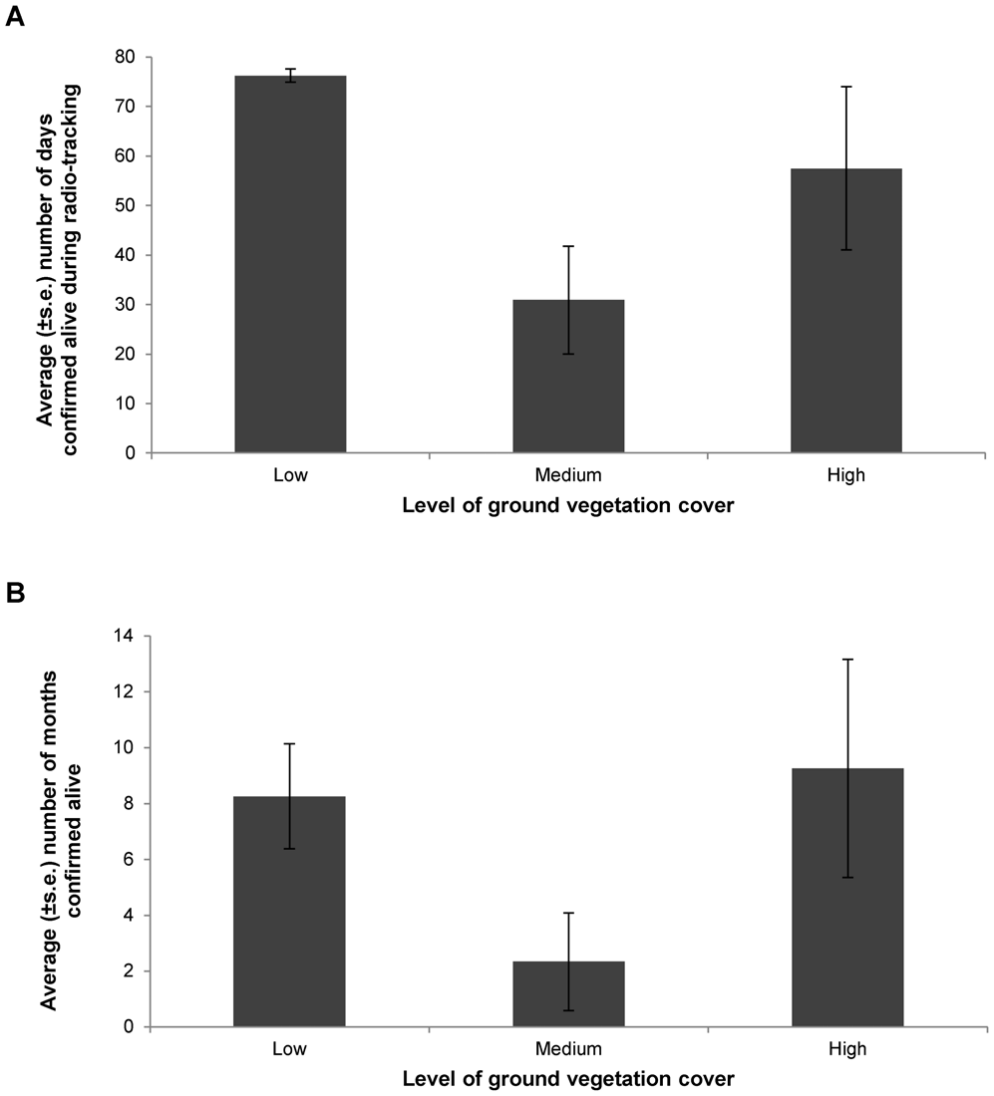
Confirmed survival. Average survival (± s.e.) for reintroduced brown treecreeper individuals: (a) the number of days confirmed alive during the radio-tracking period 16 November 2009 to 4 February 2010); and (b) the number of months confirmed alive during monitoring for 16 months after release (to March 2011). Results are given according to the level of ground vegetation cover most experienced by the individual during the monitoring period. Sample sizes of individuals are as follows: (a) Low: 8; Medium: 8; High 2; (b) Low: 8; Medium: 6; High: 4.

We detected 91% confirmed survival of released brown treecreepers 3 day post-release, 65% four weeks post-release and 42% survival six months post-release (see [30] for extensive details on reintroduced brown treecreeper survival). We detected a significant effect of ground vegetation cover on the daily survival of brown treecreepers over the radio-tracking period (χ^2^ = 11.050, d.f. = 2, P = 0.016), with high predicted survival for individuals that primarily used dry forest polygons with low levels of ground vegetation cover (Figure 3 a). However, there was not a significant influence of gender on survival (χ^2^ = 2.071, d.f. = 1, P = 0.172), or variation in survival according to social group (σ^2^ = 0.115; P = 0.098). Monthly survival of individuals over sixteen months to March 2011 was not significantly influenced by either ground vegetation cover (χ^2^ = 1.090, d.f. = 2, P = 0.614), or gender (χ^2^ = 0.050, d.f. = 1, P = 0.823), although there was significant variation due to social group (σ^2^ = 0.781; P = 0.036). For both time periods survival was lowest for individuals within woodland areas with medium levels of ground vegetation cover (Figure 3 a and b).

### Costs of Searching in Unfamiliar Habitat

The time to settlement for a social group (average 28.33 days (±5.78 s.e.)) did not significantly influence the number of group members alive at settlement (when calculated in relation to the number of group members at release) (χ^2^ = 0.140, d.f. = 1, P = 0.709).

## Discussion

We examined whether experimental treatments at the reintroduction site (specifically variations in ground vegetation cover, and installation of nest boxes) influenced the movement, selection of final home range and survival of reintroduced brown treecreepers. The key findings of our analyses were: 1) some individuals made extensive movements irrespective of the release site habitat characteristics or gender; 2) social factors appeared to influence the movement and survival of brown treecreeper individuals more than habitat; and 3) brown treecreepers showed some preference for dry forest areas, although there was only limited evidence that experimental restoration treatments influenced the selection of final home range and survival.

### Search Techniques

We observed extensive movements by reintroduced brown treecreeper individuals during the radio-tracking period. In particular, the average distance and maximum distance of forays (1550.71 m; and 7.60 km respectively) were greater than distances previously observed among brown treecreeper natal dispersers (1099 m and 2.60 km respectively) [51]. Further, the three largest home ranges (12.86 to 29.30 ha) were much greater than typically recorded elsewhere in south-east Australia (average 3–6 ha) [45]. The extensive movements that we observed also occurred in individuals of other species that have been translocated [21], [23], [66]. Extensive movements may be a result of: 1) a lack of conspecifics due to the absence of resident brown treecreepers within the release reserves, since conspecifics engaged in territory defence might encourage individuals to remain close to the release site [16], [67]; 2) the large size of the nature reserves (1623 ha of connected habitat compared to the source habitat which consisted of remnant patches linked by corridors or scattered trees and ranging in size from five to 90 ha) which reduces patch boundaries [68]; 3) low habitat quality which can be associated with larger home ranges [69], [70] (ground foraging habitat and refuge habitat is of lower quality in the reintroduction reserves in comparison to the source sites (unpublished data)); or 4) possible rejection of the release site [18]. Thus, such extensive movements may signal a problem with release sites in reintroductions. Yet, the result also indicates that reintroduced individuals are likely to be able to adjust their movement behaviours and find suitable habitat, even if it exists outside their normal dispersal distances. Extensive movement by released individuals may also result in an underestimation of true survival. However, it is unlikely that many individuals have survived undetected given that detectability of brown treecreepers with and without radio-transmitters did not differ substantially [30]. Further, it could be argued that the disappearance of individuals due to dispersal is just as much a failure of the reintroduction as mortality of individuals [71].

In our examination of the weekly search area, we did not identify a significant effect of monitoring week, but the data suggested a roughly quadratic relationship between monitoring week and search area. This is in contrast to our prediction that individuals would actively search widely through the environment before choosing to settle, thus exhibiting a decrease in search area over time. Indeed, translocated individuals, particularly birds, often display high rates of relatively immediate dispersal away from the release site [20], [66], [72], [73]. The relationship we observed between search area and week is not frequently reported and may be a result of releasing birds in social groups with familiar group members [74], or initial caution by individuals due to the translocation or inexperience in an unfamiliar environment [75], [76], followed by more active search for habitat, and then eventual settlement. This would be an advantageous approach, since previous studies have indicated that bolder individuals or those moving greater distances suffer increased mortality [19], [77], [78]. We did, however, identify highly significant variation in search area among individuals. High individual variation has previously been identified in the movement of brown treecreeper natal dispersers [54], [59], which may be influenced by the benefits of various search tactics, and hence individual variation may contribute to the unexpected results in the movement of reintroduced individuals.

Our analyses of brown treecreeper movement parameters (foray distance, foray rate, search rate and search area), showed that movement was not significantly influenced by the release site habitat quality. This was despite a priori predictions that individuals released in poorer quality habitat (postulated to be those with high levels of ground vegetation cover and no nest boxes) would be more inclined to disperse [11], [12] and hence would have increased movement (but see [13]). Thus, reintroduced individuals may always explore their surroundings regardless of the quality of the habitat they are provided with. However, the lack of an effect of the release site in our study may also be due to large individual variation [54], [59]; or the potential effects of other factors on movement such as additional habitat factors, predation pressure or body condition [15], [17], the comparison of habitat characteristics with those present in the individual’s natal site [18], or stress following translocation [79]. Additionally, movement parameters were not influenced by gender, with both males and females undertaking extensive forays. This was unexpected since natal dispersal by the brown treecreeper (and indeed by many bird species [80]) is largely female-biased [45]. This may be a particularly important result, as it suggests that movement behaviour following a reintroduction is not easily predictable, and should not be exclusively based on studies of movement in other contexts, such as natal dispersal.

We found significant variation between social groups for all analyses of brown treecreeper movement. This indicates that social factors or group characteristics may be a more important influence on movement and dispersal than habitat characteristics, although it is still unclear exactly what those social factors might be. One consequence of this finding is that regardless of how precisely a release site is chosen, some individuals and groups are still likely to move extensively.

### Habitat Attributes at Settlement and their Effect on Survival

The settlement of social groups was significantly influenced by the level of ground vegetation cover within a polygon. Settlement was highest in dry forest polygons with low levels of ground vegetation cover. However, settlement was lowest in polygons with medium ground vegetation cover rather than those with high cover. Daily survival over the radio-tracking period and monthly survival showed similar trends, with high survival in polygons with high and low ground vegetation cover, and lowest survival in polygons with medium ground vegetation cover. We had predicted that woodland areas with lower levels of ground vegetation (which would correspond to polygons with medium levels of ground vegetation cover) would be preferred based on extensive literature on the species’ requirements, which suggests that lower levels of cover increase accessibility to invertebrate prey for this woodland-dependent, ground-foraging species [38], [81] and facilitate easier detection of and escape from predators [34]. Instead, brown treecreepers showed a preference for areas with the lowest ground vegetation cover, which were dry forest areas, but they also preferred high cover over medium cover in woodland areas. These unexpected results may be influenced by factors that we did not measure, such as predation events, or the condition of the social group’s natal habitat, which may result in the rejection of suitable habitat or the selection of suboptimal habitat [18], [82]. However, areas with medium ground vegetation cover in these reserves correlated with woodland areas with more intensive kangaroo grazing and/or a history of intense livestock grazing in comparison to woodland areas with high ground vegetation cover. Although grazing may improve the accessibility of invertebrate prey [38], grazing is also likely to reduce the condition of the ground layer and decrease the abundance and diversity of the associated invertebrate prey [81], [83], [84]. This suggests that this woodland bird may actually prefer dry forests when woodlands have declined in condition, and that additional investigation of habitat preferences and restoration techniques are required. Further, previously reported tolerance of grazing in this species may be misleading, as grazing is unlikely to provide a substitute for the natural processes that would occur in woodlands to create areas of low ground vegetation cover important to the brown treecreeper such as a cryptogamic crust and dense leaf litter layer [36], [37]. Thus, a more nuanced understanding of the relationship between woodland birds and woodland vs. forest habitats may be required to reliably predict areas that will be good quality habitat for reintroductions.

We detected significantly higher settlement in polygons without nest boxes. This result was unexpected based on our predictions that individuals would be more likely to settle in polygons with nest boxes. However, this result was only slightly significant (P = 0.046), and may have been influenced by the general movement of brown treecreeper social groups to non-experimental, dry forest polygons. This result may also be influenced by other habitat characteristics such as the quantity of naturally-occurring cavities [33], which was not quantified in this study, although are known to be limiting in relation to other habitats supporting the brown treecreeper (unpublished data). We did not have an appropriate opportunity to test the use of nest boxes and did not observe any individuals utilizing the nest boxes. However, there are many existing observations of the species using artificial hollows with a wide variety of characteristics [39].

### Costs of Searching in Unfamiliar Habitat

Our analyses indicated that settlement time did not significantly influence the survival of group members at settlement. We predicted that longer settlement times for social groups would result in higher mortality of individuals due to the costs of searching within an unfamiliar environment [26], [28], [77]. It is possible that the use of exploratory forays and in particular the relatively low average foray rate employed (0.18 forays/day), allowed individuals to explore the wider environment without incurring significant costs such as an increased risk of predation. However, although our study obtained many observations on brown treecreeper movement, the logistical and financial difficulties associated with radio-tracking large numbers of individuals prevented us from obtaining enough data to conduct statistical analyses with high power. Therefore, we need to be cautious about our conclusions from this paper, such as any suggestions that searching does not entail costs. However, this study provided a unique opportunity to examine the details of movement and habitat selection of a reintroduced ground-foraging insectivore.

## Supporting Information

Information S1 **Details of the 43 individual brown treecreepers reintroduced within the seven social groups.**
(DOC)

Information S2 **Foray identification technique for reintroduced brown treecreepers.**
(DOCX)

Information S3 **Determining settlement for reintroduced brown treecreeper social groups.**
(DOCX)
