# Supplementary material for: Habitat Selection and Post-Release Movement of Reintroduced Brown Treecreeper Individuals in Restored Temperate Woodland
Source: PLoS One. 2012 Dec 5;7(12):e50612. doi: 10.1371/journal.pone.0050612 (PMC3515574; doi:10.1371/journal.pone.0050612)
Supplement: Information S1 — Details of the 43 individual brown treecreepers reintroduced within the seven social groups. (DOC) [file pone.0050612.s001.doc]

**Information S1**

**Details of the 43 individual Brown Treecreepers reintroduced within the seven social groups**

Individuals are identified by their leg-bands, the first two coloured bands were on the left leg and the remaining two on the right leg, with the uppermost band stated first. Band colours: B, dark blue; G, dark green; K, black; L, light blue; M, metal; R, red; S, split white and black; U, mauve; Y, yellow. Age is given in years and ‘F’ denotes dependent fledgling

| **Identification** | **Group** | **Sex** | **Age** | **Release Date** | **Transmitter?** |
| --- | --- | --- | --- | --- | --- |
| BLMR | 1 | M | 1+ | 16/11/2009 | Y |
| BLML | 1 | F | 1+ | 16/11/2009 | Y |
| KSMS | 1 | F | F | 16/11/2009 | N |
| RGMU | 1 | M | F | 16/11/2009 | N |
| GLMU | 2 | F | 1+ | 18/11/2009 | Y |
| BRMK | 2 | M | 1+ | 18/11/2009 | Y |
| UKMR | 2 | M | 1+ | 18/11/2009 | Y |
| RSML | 2 | M | F | 18/11/2009 | N |
| LUMY | 2 | M | F | 18/11/2009 | N |
| YYMY | 2 | M | 1+ | 18/11/2009 | N |
| SLMG | 2 | M | 1+ | 18/11/2009 | N |
| KBMS | 2 | M | F | 18/11/2009 | N |
| YGMS | 3 | F | 2+ | 23/11/2009 | Y |
| GGMB | 3 | M | 1+ | 23/11/2009 | Y |
| BUMG | 3 | M | F | 23/11/2009 | N |
| UUMK | 3 | M | 2+ | 23/11/2009 | N |
| KLMR | 3 | M | 1+ | 23/11/2009 | N |
| SUMS | 3 | M | F | 23/11/2009 | N |
| USMB | 4 | M | 2+ | 25/11/2009 | Y |
| YKMU | 4 | F | 1+ | 25/11/2009 | Y |
| RUMK | 4 | M | 1+ | 25/11/2009 | Y |
| SGMU | 4 | M | 2+ | 25/11/2009 | N |
| BRMY | 4 | M | F | 25/11/2009 | N |
| LUMR | 4 | F | F | 25/11/2009 | N |
| LGMS | 5 | F | 1+ | 27/11/2009 | Y |
| BKMK | 5 | F | F | 27/11/2009 | N |
| SRMG | 5 | M | 1+ | 27/11/2009 | N |
| GBMR | 5 | F | 1+ | 27/11/2009 | Y |
| SLMY | 5 | F | F | 27/11/2009 | N |
| YLMG | 5 | M | 2+ | 27/11/2009 | N |
| RRMU | 5 | M | 1+ | 27/11/2009 | Y |
| KSMB | 5 | M | 2+ | 27/11/2009 | N |
| KGMG | 6 | F | 2+ | 29/11/2009 | Y |
| RGMB | 6 | M | 2+ | 29/11/2009 | Y |
| SUML | 6 | M | F | 29/11/2009 | N |
| BLMU | 6 | M | F | 29/11/2009 | N |
| GBMS | 6 | F | F | 29/11/2009 | N |
| UBMR | 7 | M | 1+ | 1/12/2009 | Y |
| LSMU | 7 | M | 2+ | 1/12/2009 | Y |
| RSMB | 7 | F | 2+ | 1/12/2009 | Y |
| BKMY | 7 | F | F | 1/12/2009 | N |
| YRMY | 7 | M | F | 1/12/2009 | N |
| GLMS | 7 | M | F | 1/12/2009 | N |
