# Supplementary material for: Habitat Selection and Post-Release Movement of Reintroduced Brown Treecreeper Individuals in Restored Temperate Woodland
Source: PLoS One. 2012 Dec 5;7(12):e50612. doi: 10.1371/journal.pone.0050612 (PMC3515574; doi:10.1371/journal.pone.0050612)
Supplement: Information S2 — Foray identification technique for reintroduced brown treecreepers. (DOCX) [file pone.0050612.s002.docx]

**Information S2**

**Foray identification technique for reintroduced brown treecreepers**

To distinguish forays from normal movements, we first needed to determine the usual range of movement by reintroduced brown treecreeper individuals. We used Hawth’s Analysis Tools version 3.27 within ESRI® ArcmapTM 9.2 to plot 50 m buffer zones (to reflect an assessment radius) around each point where an individual was located (Figure 1). The assessment radius replicates the idea than an individual can assess the quality of the habitat within the assessment radius as they move through the environment. The 50 m distance was chosen based on prior observations of response to habitat features and an approaching observer [[1](#_ENREF_1)]. This approach has been used successfully in other studies and one key point is that the results are used in a relative sense, to compare among individuals, so the precision of this estimate is not critical [[2](#_ENREF_2)]. If 10 or more assessment radii overlapped, with each radius overlapping with at least five other radii, we defined this as a ‘temporary home range’. We measured north-south and east-west diameters of each temporary home range to calculate the average length of a temporary home range for each radio-tracked individual. We then defined a foray as a movement between two temporary home ranges (or away from and then back to the same temporary home range) that included at least two consecutive points separated by a distance greater than 1.5 times the average length of a temporary home range. As prior research suggests that individuals usually undertake forays alone but usually interact with conspecifics when in their home ranges [[1](#_ENREF_1)], we also used behavioural observations of birds at these locations to qualitatively verify that this approach was distinguishing between forays and home range movements.

**Information S2 Figure legends:**

**(for Figure SI 2 – 1)**

**Figure 1. Foray identification technique**

Movement paths of breeding female brown treecreepers monitored via radio-tracking. Black dots are point locations where the individual was identified, grey lines connect the consecutive locations, and circles represent a 50 m assessment radius around each location. The limits of the temporary home ranges are indicated by the thick black lines. Black lines indicate the polygons stratifying the reserves. (a) Breeding female (YKMU) radio-tracked for 58 days undertook 10 forays. Information for locations for some of the forays for this individual are as follows: Foray 1: locations from 11:05 to 17:50 6/12/2009; Foray 2: locations from 15:28 12/12/2009 to 15:15 13/12/2009; Foray 3: locations from 11:45 to 17:04 16/12/2009; and Foray 4: locations from 15:21 18/12/2009 to 13:10 22/12/2009. (b) Breeding female (LBML) radio-tracked for 70 days and did not undertake any forays.

**References**

1. Doerr ED, Doerr VAJ (2005) Dispersal range analysis: quantifying individual variation in dispersal behaviour. Oecologia 142: 1-10.

2. Doerr VAJ, Doerr ED (2004) Fractal analysis can explain individual variation in dispersal search paths. Ecology 85: 1428-1438.
