# Supplementary material for: Habitat Selection and Post-Release Movement of Reintroduced Brown Treecreeper Individuals in Restored Temperate Woodland
Source: PLoS One. 2012 Dec 5;7(12):e50612. doi: 10.1371/journal.pone.0050612 (PMC3515574; doi:10.1371/journal.pone.0050612)
Supplement: Information S3 — Determining settlement for reintroduced brown treecreeper social groups. (DOCX) [file pone.0050612.s003.docx]

**Information S3**

**Determining settlement for reintroduced brown treecreeper social groups**

To determine the point location at which a social group had settled (and subsequently the time to settlement), we analysed the movement of the breeding female as a representative for the group, except where the female was deceased prior to settlement in which case the breeding male was used. For each point that an individual was located during the tracking period, we calculated the distance between that point and the final location for that individual. We graphed these distances against location number for each individual (see Figures 1 - 6 below). An individual was recognised as having settled when the distance to final location reached a lower asymptote. This was guided by the assumption that early explorations might be far from the final home range, but once settlement occurs locations should all be within a home range’s diameter of each other. Hence the distance to the final location would decrease and then remain relatively constant. Once the point location of settlement was determined, we could then identify the time at which this point location was recorded. Comparison with the release time, we could then calculate time to settlement. Note that all members of social group 5 were either deceased or had disappeared before settlement.

**Information S3: Figure Captions**

**(for Figure SI3 – 1 to Figure SI3 – 6)**

**Figure 1. Determination of settlement for social group 1**

Based on the movement of Lobelia (LBML), breeding female. Settlement at location 55 after 24 days.

**Figure 2. Determination of settlement for social group 2**

Based the movement of Glue (GLMU), breeding female. Settlement at location 57 after 27 days.

**Figure 3. Determination of settlement for social group 3**

Based on the movement of Yagis (YGMS), breeding female. Settlement at location 10 after 5 days.

**Figure 4. Determination of settlement for social group 4**

Based on the movement of Haiku (YKMU), breeding female. Settlement at location 58 after 28 days.

**Figure 5. Determination of settlement for social group 6**

Based on the movement of Kaja Goo Goo (KGMG), breeding female. Settlement at location 100 after 41 days.

**Figure 6. Determination of settlement for social group 7**

Based on the movement of Uber (UBMR), breeding male. Settlement at location 115 after 45 days.
